# Controlled access to lumasiran in primary hyperoxaluria type 1: evaluation of a new access route for orphan drugs in the Netherlands

**DOI:** 10.1093/ndt/gfaf060

**Published:** 2025-03-22

**Authors:** Lisa J Deesker, Casper F M Franssen, Eiske Dorresteijn, Nicole C A J van de Kar, S Azam Nurmohamed, David Severs, Sander F Garrelfs, Anke A M G Pisters-van Roy, Carla E M Hollak, Jaap W Groothoff

**Affiliations:** Department of Pediatric Nephrology, Emma Children's Hospital, Amsterdam UMC, University of Amsterdam, Amsterdam, The Netherlands; Department of Nephrology, University Medical Center Groningen, University of Groningen, Groningen, The Netherlands; Department of Pediatric Nephrology, Sophia Children's Hospital, Erasmus Medical Center, Rotterdam, Netherlands; Radboud University Medical Center, Amalia Children's Hospital, Radboud Institute for Molecular Life Sciences, Department of Pediatric Nephrology, Nijmegen, The Netherlands; Department of Nephrology, Amsterdam University Medical Center, Amsterdam, The Netherlands; Department of Internal Medicine, Division of Nephrology and Transplantation, Erasmus Medical Center, Rotterdam, The Netherlands; Department of Pediatric Nephrology, Emma Children's Hospital, Amsterdam UMC, University of Amsterdam, Amsterdam, The Netherlands; Department Medical Policy and Advice, CZ Healthcare Insurance, The Netherlands; Department of Endocrinology and Metabolism, Amsterdam UMC location University of Amsterdam, Amsterdam, The Netherlands; Medicines for Society, Platform at Amsterdam UMC location University of Amsterdam, Amsterdam, The Netherlands; Department of Pediatric Nephrology, Emma Children's Hospital, Amsterdam UMC, University of Amsterdam, Amsterdam, The Netherlands

**Keywords:** controlled access model, lumasiran, orphan drugs, PH1, primary hyperoxaluria

## Abstract

**Background and hypothesis:**

In search for controlled access to expensive innovative orphan drugs, a national access route called ‘Orphan Drug Access Protocol’ (ODAP) was developed and piloted with lumasiran, a new drug for patients with primary hyperoxaluria type 1 (PH1). Here, we present a 2-year evaluation of this pilot study.

**Methods:**

Specialists from the Dutch PH1 Expert Centre and the national ODAP steering group developed a protocol for controlled and conditional treatment of children and adults with PH1 with lumasiran. Indication for treatment is based on specific clinical characteristics. Cessation or continuation of therapy is evaluated every 6 months for 5 years by a national indication committee consisting of PH1 specialists, based on biochemical and clinical response. Drug wastage is minimized by centralizing and pooling patients for administration.

**Results:**

Between September 2022 and September 2024, 21 PH1 patients were reviewed and 76% were deemed eligible for lumasiran treatment. Ten patients were already receiving lumasiran through clinical trials or early access programs at time of assessment. The follow-up time with lumasiran was 0.1–6.6 years, including trial years. All patients with >1 year lumasiran treatment responded significantly biochemically and clinically. Details on outcomes are presented. Denials for lumasiran therapy were nearly all based on full pyridoxine responsiveness. All denied patients, except one, had good clinical outcomes. This patient received lumasiran after initial denial based on clinical and biochemical course. Patient selection and minimizing wastage saved approximately €3 227 065 per year based on the official list price.

**Conclusions:**

This national ODAP protocol enabled access to lumasiran therapy for severely ill patients, prevented unnecessary treatment in others, and provided new insights into the real-world effectiveness of lumasiran in PH1 patients through systematic monitoring. It may serve as a template for future access routes to new expensive therapeutics in orphan diseases.

KEY LEARNING POINTS **What was known:**

- Primary hyperoxaluria type 1 (PH1) is a rare genetic disorder with a severe heterogeneous course that requires personalized and expert therapeutic management.
- Lumasiran was approved by the European Medicines Agency in 2020, but its clinical effectiveness and role in pyridoxine responsive patients is not fully understood.
- Limited patient numbers, high costs and uncertainty about the exact clinical benefit over time have hampered rapid reimbursement of lumasiran by national health systems in many countries, leading to significant global disparities in access to this potentially effective drug, even within European countries.

**This study adds:**

- The national Orphan Drug Access Protocol (ODAP) provided rapid access to lumasiran therapy for PH1 patients who may benefit most. It prevented unnecessary treatment in others, and provided new insights in the use of lumasiran, while minimizing costs.
- Centralized and standardized monitoring in the ODAP allowed the collection of reliable real-world data, clinical data on effectiveness and side effects over a longer period, ensuring a reliable assessment of therapeutic outcomes. This standardized approach by a team of multiple experts distinguishes this approach from standard expert centre monitoring.
- Within the ODAP, patients received high quality, personalized care through collaboration between local nephrologists and a national network of experts in the field of primary hyperoxaluria.

**Potential impact:**

- Controlled access strategies such as ODAP offers a scalable model for managing rare kidney diseases, ensuring equitable access, close monitoring and high-quality data collection.
- It highlights the importance of expert-led, centralized protocols to achieve optimal patient care and generate actionable insights in rare disorders.

## INTRODUCTION

Primary hyperoxaluria type 1 (PH1) is a rare inherited metabolic disease characterized by endogenous overproduction of oxalate due to deficiency of the liver-specific peroxisomal enzyme alanine:glyoxylate aminotransferase (AGT), leading to kidney stones, nephrocalcinosis and kidney failure in over 60% of patients. PH1 patients with kidney failure are at risk for life-threatening systemic disease due to oxalate depositions in several tissues. To date, liver transplantation is the only cure. Lumasiran, an RNA interfering (RNAi) therapy, blocks the production of the enzyme glycolate oxidase, thereby reducing the production of glyoxylate, the substrate for oxalate. It was approved by both the Federal Drug Agency (FDA) and European Medicines Agency (EMA) in November 2020 for PH1 based on promising results from a phase 3 trial [1]. However, limited patient numbers, high costs and uncertainty about the exact clinical benefit over time have hampered rapid reimbursement by national health systems in many countries, leading to significant global disparities in access to this potentially effective drug, even within European countries [2].

The situation of lumasiran in PH1 is not unique. A small target group of patients with great clinical diversity, uncertainty about real world long-term clinical benefits at time of introduction and high costs are well-known barriers to the introduction of many promising expensive orphan drugs [3]. All these issues limit knowledge about who exactly to treat and when, within the approved indication. High costs often require lengthy price negotiations, which can further limit and delay access to treatment [4]. This situation has urged stakeholders including clinicians, health insurers and patient representatives to develop a protocol for conditional and controlled access to orphan drugs, known as the Orphan Drug Access Protocol (ODAP). The aim of ODAP is to ensure relatively rapid access and payment for selected patients with an unmet need whose clinical characteristics suggest they may benefit most, while collecting additional standardized data to monitor real-world, clinical effectiveness and side effects over a longer period. This will allow promising drugs to reach patients earlier and in a more controlled way. Lumasiran was chosen as a pilot project for an ODAP. Here, we present the first 2-year evaluation of the lumasiran ODAP pilot study.

## MATERIALS AND METHODS

The national ODAP steering group developed a protocol for the controlled and conditional treatment of children and adults with PH1 with lumasiran, together with specialists from Amsterdam UMC, the expert centre for PH within the Netherlands which participated in the clinical trials for lumasiran. The protocol contains information about the disease, the position of the new drug compared with other therapies, start and stop criteria, and evaluation criteria. An indication committee was set up, including one paediatric and one adult nephrologist from each academic medical centre in the Netherlands. PH1 patients potentially eligible for lumasiran treatment are presented to an executive committee consisting of six members of the indication committee (all adult or paediatric nephrologists with experience with PH1). All work performed by the indication committee (approximately 1 h per month) was on voluntary basis. The patient's characteristics are discussed at a monthly meeting and a unanimous decision is required for positive recommendation. The administration of lumasiran is confined to the expert centre in Amsterdam, with two or more patients being clustered to limit drug wastage. Patient visits were conducted by a research nurse and PhD student.

The protocol for this pilot was based on the latest international guidelines for primary hyperoxaluria [5], including the distinction between PH1 patients with and without pyridoxine responsiveness. Based on the clinical and biochemical patient characteristics, a decision is made on initiating lumasiran within the ODAP protocol (see Table 1 with start and stop criteria). To receive treatment, patients must enrol in ODAP and agree to regular medical data collection to monitor the safety and effectiveness of treatment in accordance with the local regulations and with approval of the Institutional Review Board of Amsterdam UMC and must not have received a successful liver transplantation. Patients are monitored by recommended measurements [6], including kidney function, vital signs, urine and plasma oxalate, kidney and urinary tract ultrasound and screening for systemic oxalosis. All patient data are captured within the OxalEurope database via the GCP-proof online platform Castor EDC. Patients are required to visit Amsterdam UMC every 3 months for subcutaneous lumasiran administration and monitoring. The treatment dose is in line with the recommended dosage (Supplementary data, Table S1). Minor modifications have been made to the protocol over time based on the committee's experience (e.g. the use of drugs other than lumasiran has been explicitly stated).

**Table 1:** Start and stop criteria in the ODAP.

	Start	First analysis (after 6 months) and stop criteria	6-montly analysis (up to 5 years) and stop criteria
Group A: eGFR >30 mL/min/1.73 m^2^, B6 unresponsiveness or partial responsive and clinical manifestations or age <18 years	Unconditionally	Uox >1.5 ULN OR <30% reduction OR deterioration clinical condition as assessed by committee OR SAE	6 monthly: SAE OR deterioration clinical condition, potentially due to lumasiran
Group B: eGFR >30 mL/min/1.73 m^2^, B6 responsive and clinical manifestations	After discussion on clinical data	Uox >1.5 ULN OR <30% reduction OR deterioration clinical condition as assessed by committee OR SAE^a^	6 monthly: SAE AND/OR deterioration clinical condition, potentially due to lumasiran
Group C: eGFR <30 mL/min/1.73 m^2^, genetic variant consistent with B6 unresponsiveness	After definition monitoring Pox based on clinical data	No expected rate of decrease Pox^b^ OR deterioration clinical condition as assessed by committee OR SAE^a^	6 monthly: no decrease >50% Pox, if less discussion yearly: SAE OR deterioration clinical condition, potentially due to lumasiran
Group D: eGFR <30 mL/min/1.73 m^2^, genetic variant consistent with B6 responsiveness	After definition monitoring Pox based on clinical data after 3 month observation under full B6 therapy	No expected rate of decrease Pox^b^ OR deterioration clinical condition as assessed by committee OR SAE	6 monthly: no decrease >50% Pox,^c^ if less discussion yearly: SAE OR deterioration clinical condition, potentially due to lumasiran
Group E: Renal failure with unknown etiology and suspicion PH1	Start lumasiran—monthly monitoring plasma oxalate levels	In case of no response on plasma oxalate levels after 6 months OR type 2 or 3 genetically assessed, or SAE	Not applicable

^a^Including unexpected decline in kidney function, not related to another cause than hyperoxaluria. Deterioration should be evaluated in the context of the individual patient and may include unexpected decline in kidney function, not related to another cause than hyperoxaluria and/or worsening of nephrocalcinosis (e.g. occurrence of new kidney stones as detected by ultrasound; recurrent attacks due to pre-existing stones are no criterion for failure).

^b^In case of no kidney transplantation; in case of kidney transplantation continuation lumasiran in case of normalization of Pox within 3 months after kidney transplantation.

^c^In case of no kidney transplantation; in case of kidney transplantation stop lumasiran 3 months after kidney transplantation in case of normalization of urinary oxalate excretion, repeated measurement Uox every month, restart lumasiran in case of increase Uox >1.0 ULN.

B6: vitamin B6, supplemented as pyridoxine; Uox: urinary oxalate; SAE: severe adverse event as defined by the Central Committee on Research Involving Human Subjects [21].

**Figure 1:**
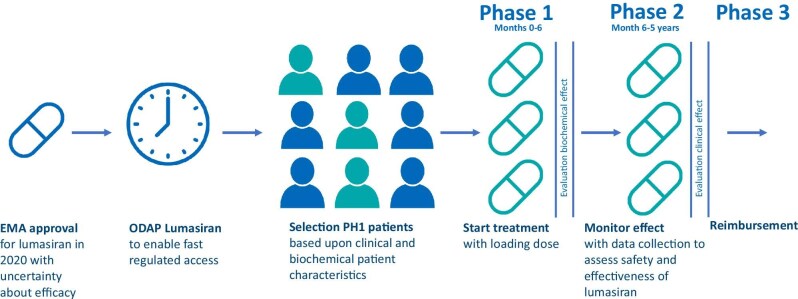
Overview of ODAP protocol.

The pilot protocol consists of three phases following approval of the initiation of lumasiran in an individual patient (Fig. 1). In phase 1 (Months 0–6), the effectiveness of the drug in an individual patient is assessed based on biochemical effects (urinary and plasma oxalate). In phase 2 (Month 6 to 5 years), the therapeutic effect is monitored based on the clinical condition as evaluated by the indication committee of paediatric and adult nephrologists according to predefined criteria (see Table 1 for all criteria) based on the different patient categories (groups A–E), further specified in Table 1. Different confidential price agreements apply between health insurers and manufacturers in these two phases, which represent the certainty about the effectiveness of the drug per phase [4]. There are no out-of-pocket costs for the patients in either phase. After 5 years of decision making via the ODAP, final data will be analysed for therapeutic effectiveness and the health insurers will decide whether lumasiran will be reimbursed permanently for patients, referred to as phase 3.

For the analyses reported in this manuscript (including Table 2), mean urinary oxalate and plasma oxalate levels at follow-up were calculated as the mean of measurements >6 months after initiation of lumasiran until the last follow-up measurement. Oxalate reduction is calculated as the percentage reduction between baseline (last measurement before start lumasiran) and mean follow-up level as described above. Normalization of urinary oxalate was defined as <0.5 mmol/24 h/1.73 m^2^ or oxalate-to-creatinine ratio ≤0.06 mmol/mmol and plasma oxalate as <6.8 µmol/L. All measurements were performed in the same laboratory (i.e. Amsterdam UMC) [7]. Cost savings were calculated using the Dutch list price (exact prices have not been made public within the Netherlands) and verified with data from the Canadian Agency for Drugs and Technologies in Health (Supplementary data, Table S2).

**Table 2:** Patient characteristics of patients found eligible for treatment with lumasiran.

	Patient	Genotype	Age at diagnosis (years)	Age at start lumasiran or ODAP evaluation (years)	Trial or EAP before ODAP	Clinical symptoms at start lumasiran or ODAP evaluation	eGFR at start lumasiran or ODAP evaluation	UOx or POx at onset lumasiran or ODAP evaluation and at FU	Years since start lumasiran/ODAP	% decrease in oxalate	Clinical symptoms at FU	Effect lumasiran^a^
Group A: eGFR >30 mL/min/1.73 m^2^, B6–	1	c.454T>A and c.508G>A	5	35	Clinical trial, start phase 2	Stone events, urological procedures, urinary tract infections, nephrocalcinosis, CKD	Start: 53, FU: 47	UOx start: 0.19, UOx FU: 0.08	6.6/1.9	–58	No stone events, decreased frequency of urinary tract infections	Biochemical effect, no normalization
	2	Exon 5–11 deletion and Exon 5–11 deletion	3	31	Clinical trial, start phase 2	Stone events, urological procedures, nephrocalcinosis, CKD	Start: 77, FU: 80	UOx start: 0.17, UOx FU: 0.05	6.5/1.7	–71	One stone event 2.5 years after start lumasiran, stable kidney function, injection site reaction	Normalization
	3	c.33insC and c.33insC	5	15	Early access, start phase 2	Stone events, nephrocalcinosis,	Start: >90, FU: >90	UOx start: 0.12, UOx FU: 0.05	3.4/0.7	–62	Asymptomatic, no stone events, stable kidney function	Normalization
	4	c.33delC and c.454T>A	3	25	Clinical trial, start phase 2	Frequent stone events with urological procedures, frequent urinary tract infections, nephrocalcinosis	Start: 57, FU: 51	UOx start: 0.16, UOx FU: 0.08	5.3/0.7	–48	Residual stones, no stone events, stable nephrocalcinosis and kidney function, injection site reaction	Biochemical effect, no normalization
	5	c.33delC and c.508G>A	4	7	Clinical trial, start phase 2	Stone events, frequent urological procedures, nephrocalcinosis	Start: 90, FU: 86	UOx start: 0.55, UOx FU: 0.09	5.3/0.7	–84	Asymptomatic, stable nephrocalcinosis	Biochemical effect, no normalization
	6	c.33insC and c.508G>A	5	19	Clinical trial, start phase 2	Stone events, nephrocalcinosis,	Start: 74, FU: 78	UOx start: 0.12, UOx FU: 0.07	5.3/0.7	–44	Asymptomatic, stable kidney function	Biochemical effect, no normalization
	7	c.33insC and c.454T>A	8	20	Clinical trial, start phase 2	Stone events, frequent urological procedures, nephrocalcinosis	Start: 67, FU: 69	UOx start: 0.17, UOx FU: 0.05	5.3/0.7	–68	Stable nephrocalcinosis, 2 episodes of urinary tract infection, none in the last year	Normalization
Group B: eGFR >30 mL/min/1.73 m^2^, B6+	8	c.508G>A and c.508G>A	17	26		Stone events, frequent urological procedures	Start: 57, FU: 63	UOx start: 0.19, UOx FU: 0.07	0.9/0.9	–63	No stone events, injection site reaction	Biochemical effect, no normalization/suboptimal
	9	c.508G>A and c.508G>A	3	8	Clinical trial, start phase 2	Stone events, nephrocalcinosis,	Start: >90, FU: >90	UOx start: 0.39, UOx FU: 0.07	5.3/0.7	–81	Asymptomatic, stable nephrocalcinosis	Biochemical effect, no normalization
	10	c.508G>A and c.731T>C	0.5	23		Kidney stones	Start: >90, FU: >90	UOx start: 0.03	1.8	n.a.	Stable kidney function, no stone events, no nephrocalcinosis	Denied^b^
	11	c.731T>C and c.731T>C	0.3	6		Asymptomatic	Start: >90, FU: >90	UOx start: 0.15	0.5	n.a.	Asymptomatic	Denied^b^
Group C: eGFR <30 mL/min/1.73 m^2^, B6–	12	c.33dupC and c.33dupC; c.1075_1089 deletion	5	5	Early access, start phase 2	Kidney failure, systemic oxalosis	Start: HD/PD (<5), FU: 51, 68 after transplant	POx start: 218, POx FU: 103^c^/55^d^/10^e^	3.3/1.4	–53	Received solo kidney transplantation with stable kidney function, recurrence of nephrocalcinosis in kidney graft	Biochemical effect, no normalization
	13	c.244G>C and c.244G>C	0	35	Early access, start phase 2	Stone events, kidney failure, urological procedures, nephrocalcinosis	Start: HD (<5), FU: <5	POx start: 171, POx FU: 119	2.4/1.9	–30	Decreased systemic oxalosis under lumasiran, liver transplantation.	Biochemical effect, no normalization/suboptimal
	14	33insC and c.508G>A	54	68		LKTx and 2 solo kidney transplantations, all with oxalate nephropathy	Start: 20 FU: <5	POx start: 36	1.9	n.a.	Failure of most recent kidney graft	Denied^b^
Group D: eGFR <30 mL/min/1.73 m^2^, B6+	15	c.454T>A and c.508G>A	46	69		Stone events, nephrocalcinosis, CKD 5	Start: 13, FU: 13	UOx start: 0.11, POx start: 31, UOx FU: 0.07, POx FU: 22	0.1/0.1	–36 (u)/–27 (p)	Asymptomatic	Pending
	16	c.454T>A and c.508G>A	65	67		Stone events, kidney failure	Start: 10, FU: 78	UOx start: 0.07, POx start: 36	1.9	n.a.	Solo kidney transplantation with stable kidney function	Denied^b^
Group E:^f^ Renal failure with unknown etiology	17	c.33dupC and c.33dupC; c.481G>A	0.4	0.4		Kidney failure, systemic oxalosis	Start: HD/PD (<5), FU: 68	POx start: 214, POx FU: 73	1.1/1.1	–66	Stable kidney function, calcium oxalate crystals on kidney graft biopsy after solo kidney transplantation	Biochemical effect, no normalization

All ages and periods are presented in years.

^a^Classification of lumasiran effect with either normalization; significant biochemical effect, but no normalization; or suboptimal or no effect.

^b^This patient was denied for lumasiran treatment.

^c^POx level with lumasiran treatment,

^d^POx level with lumasiran and nedosiran treatment,

^e^POx level with lumasiran and after kidney transplantation.

^f^This patient rolled over into group C after definitive diagnosis.

CKD: chronic kidney disease; EAP: early access program; FU: follow-up; UOx: urinary oxalate level in mmol/mmol creatinine in 24-h urine except patient 17, in which spot urines were gathered; POx: plasma oxalate level in µmol/L; eGFR: estimated glomerular filtration rate in mL/min/1.73 m^2^; B6: vitamin B6 responsive (+) or unresponsiveness (–), supplemented as pyridoxine; n.a.: not applicable; LKTx: combined liver–kidney transplantation.

Medication was prepared using a vial sharing protocol after review of the product by a pharmacist of the Amsterdam UMC, leading to the recommendation that lumasiran vials may be used for multiple doses within 24 h after opening using aseptic procedures (see Supplementary data, Methods S3).

## RESULTS

**Figure 2:**
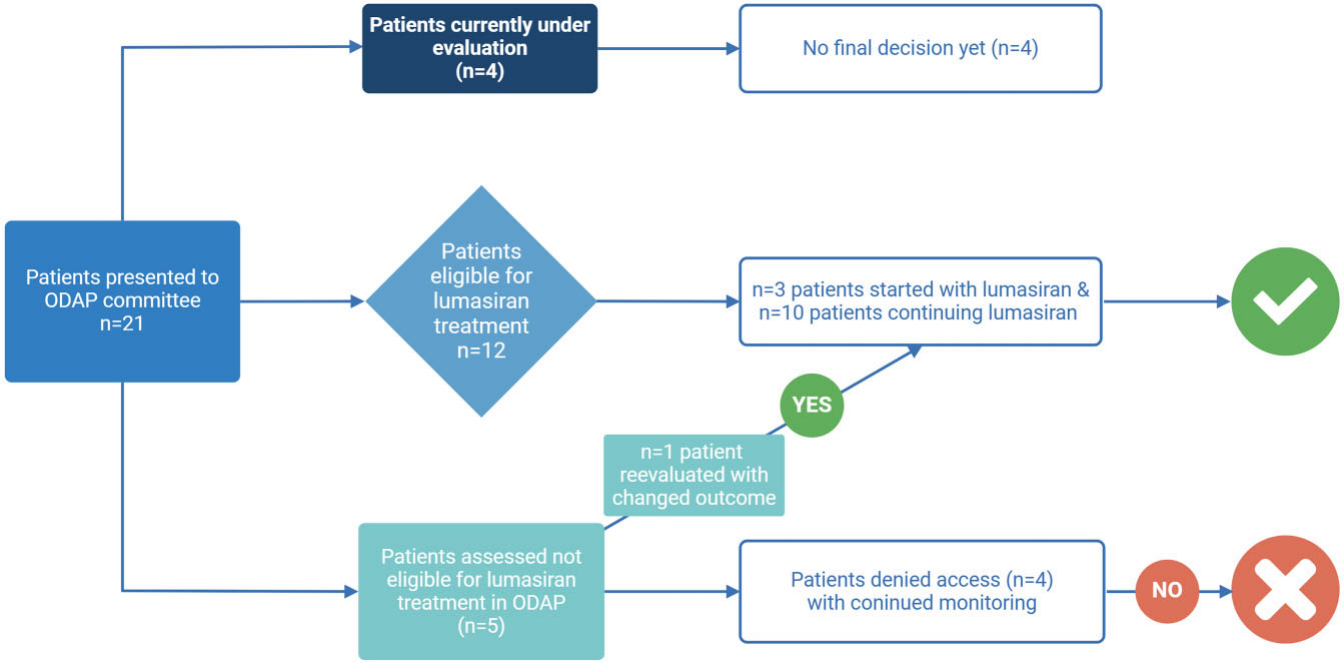
Flow diagram of patients in ODAP protocol.

Between 1 September 2022 and 1 September 2024, 21 PH1 patients were reviewed by the committee (Table 2). Thirteen met the start criteria for initiation of lumasiran treatment (Fig. 2). Reasons for non-eligibility included sufficient response to pyridoxine treatment (*n* = 3) and having undergone a successful liver transplantation (*n* = 1). Four patients were still under evaluation at the last follow-up, as follow-up information was required to make a final decision. Seven patients transitioned from the ILLUMINATE A trial (A Phase 3 Randomized, Double-blind, Placebo-Controlled Study with an Extended Dosing Period to Evaluate the Efficacy and Safety of Lumasiran in Children and Adults with Primary Hyperoxaluria Type 1) and ILLUMINATE C trial (A Single Arm Study to Evaluate Efficacy, Safety, Pharmacokinetics, and Pharmacodynamics of Lumasiran in Patients With Advanced Primary Hyperoxaluria Type 1 (PH1)) [1, 8], phase 3 trials with lumasiran, after completing the trial and extension period and three other patients transitioned from the early access program into phase 2 of the ODAP without treatment interruption. Three patients were RNAi-naïve and started with lumasiran as part of phase 1 of the ODAP. Of 17 patients with definitive decisions, 13 (76%) were approved for lumasiran, including three out of seven (42%) RNAi-naive patients. All treated patients were compliant with protocol visits, with follow-up times within the ODAP ranging from 0.1 to 1.9 years (Table 2).

### Outcomes

Group A: 7 out of 17 patients with definitive decisions had an estimated glomerular filtration rate (eGFR) >30 mL/min/1.73 m^2^ and a pathogenic variant consistent with no or insufficient pyridoxine responsiveness. All were found eligible for treatment with lumasiran and entered phase 2 directly as they transitioned from an ILLUMINATE trial or early access program and had previously shown good biochemical and clinical response. The mean reduction in urinary oxalate before ODAP enrolment aligned with follow-up outcomes during ODAP (0%–4% difference). At last follow-up, three out of seven patients had normalized urinary oxalate levels while four had levels <1.5× the upper limit of normal (ULN). The eGFR remained stable over time (i.e. the largest decline was 6 mL/min/1.73 m^2^ over 5 years, patient 4, Table 2). All patients reported notably fewer stone events and urinary tract infections after 6 months of lumasiran treatment until the last moment of follow-up.

Group B: 4 out of 17 patients with definitive decisions had an eGFR >30 mL/min/1.73 m^2^ and homozygous pyridoxine responsive pathogenic variant (i.e. c.508G>A, c.454T>A or c.731T>C). Two patients were denied for lumasiran due to a good response to pyridoxine, maintaining stable kidney function and remaining stone-free during follow-ups of 1.8 and 0.5 years. Two other patients were approved for lumasiran. One patient (patient 8, Table 2) was initially denied access to lumasiran due to pyridoxine responsiveness with near normal urinary oxalate levels and paucity of clinical symptoms, but later developed an obstructive stone episode with kidney function decline and increased oxalate excretion (4× ULN), leading to lumasiran initiation. Follow-up will determine if the oxalate rise was genuine or stone-related. The second patient (patient 9, Table 2) was approved for lumasiran after transitioning from an ILLUMINATE trial. Despite being homozygous for the c.508G>A variant, she had persistent hyperoxaluria with insufficient pyridoxine response. She showed good response to lumasiran.

Group C/E: 4 out of 17 patients with definitive decisions presented with kidney failure and had a pathogenic variant not associated with pyridoxine responsiveness. Two were previously treated in the early access program and entered phase 2. The third patient, a 4-month-old infant, was treated within 1 week of suspected PH1 based on plasma oxalate and glycolate levels (group E, patient 17), and the diagnosis was later confirmed genetically. All three patients underwent kidney transplantation, though in two cases, lumasiran alone was insufficient to reach safe values for kidney transplantation. In one patient, lumasiran was combined with nedosiran, another RNAi therapeutic, and one patient received a liver transplantation (both patients are described in more detail elsewhere: Deesker *et al*., 2024, submitted for publication [9]). One patient was denied (patient 14, Table 2) lumasiran therapy despite hyperoxaluria and a low eGFR from his second kidney graft because he had already undergone a liver transplant. The hyperoxaluria was considered due to previously stored systemic oxalate.

Group D: 2 out of 17 patients with definitive decisions had an eGFR <30 mL/min/1.73 m^2^ and a compound heterozygous c.508G>A and c.454T>A variant. One was denied for lumasiran due to a good response to pyridoxine therapy, despite the low eGFR, which was a result of late diagnosis. He received a successful solo kidney transplantation with an eGFR of 78 and normal urinary oxalate excretion 2 years later (patient 16, Table 2). The second patient was approved for lumasiran as the plasma oxalate levels were considered too high for kidney transplantation with pyridoxine treatment; the response on lumasiran therapy for this patient is still under evaluation.

Overall, total follow-up (including treatment during the trial and early access program) of lumasiran treated patients showed a mean decrease of urinary oxalate levels of 44%–84% in patients with eGFR >30 and 30%–66% in plasma oxalate levels in patients with eGFR <30 mL/min/1.73 m^2^. All RNAi-naïve patients prior to ODAP showed a good biochemical response to lumasiran at last follow-up (with –63% in urine in patient 8, and –27% and –66% in plasma in patients 12 and 13), but no normalization. Three patients reported transient injection site reactions. No other side-effects were reported. No infectious outbreak has occurred. At the 2-year assessment, 12 of 13 patients were in phase 2; one patient was in phase 1 as she was only enrolled for 2 months at the time of the data cut-off. Except for the patient who underwent liver transplantation, none of the patients met pre-specified stop criteria and discontinued lumasiran treatment. Additional clinical data are shown in Table 2.

### Cost reduction

Three out of the four denied patients (one paediatric and two adults) would have been eligible for treatment in some other European countries. The estimated treatment costs of the denied patients would have been €1 746 756–2 620 134 per year (subsequent years versus first year of treatment, see Supplementary data, Table S2 for details), resulting in an estimated cost reduction during the current 2-year ODAP trajectory of €2 953 201. Similarly, by minimizing spillage, approximately five drug vials are saved every 3 months based on the number of patients at the end of the 2-year ODAP follow-up, resulting in approximately €1 480 309 euros that can be saved each year (Supplementary data, Table S3). Travel time to the Amsterdam UMC was <2 h one-way for all patients.

## DISCUSSION

Here, we present the 2-year outcomes of the implementation of a novel orphan drug access program for lumasiran for the treatment of PH1. In summary, the protocol ensured rapid access within 1 week and personalized management for severely ill PH1 patients, provided new insights into the real-world effectiveness of the drug in selected patients, and led to a substantial reduction of costs due to controlled access, only treating patients in need of lumasiran. All, but one patient with a denial for lumasiran treatment based on clinically established good B6 responsiveness had a good clinical outcome, while in one patient, lumasiran was initiated after re-evaluation thanks to close monitoring.

### Opportunity for rapid access

Within Europe, large variation is observed in the time to reimbursement and therefore access to new costly therapies [10]. In some countries, initial access is granted to all medicines that receive marketing authorization, followed by analysis and appraisal afterwards, while in other member states lengthy analysis and negotiation may take place before a decision is made on reimbursement. In the Netherlands, conditional access to lumasiran was provided starting 1 September 2022. In contrast, as access was not granted in the UK and Belgium until April and June 2023, respectively, and lumasiran is unavailable in some other European countries (Deesker *et al*., *Nephrology Dialysis Transplantation*, in press [11, 12]). The rapid access provided by ODAP in the Netherlands has benefited an infantile patient with severe manifestations of PH1, who started lumasiran treatment within 1 week of diagnosis. Previous experience with the lumasiran early access program showed a 1-month treatment delay due to regulatory assessments (unpublished data). Prompt initiation is crucial in PH1 patients, especially those with kidney failure, to prevent oxalate accumulation. The ODAP helped avoid such delay and ensured continued access to lumasiran.

### Personalized treatment

The protocol ensured optimal personalized care for all PH1 patients, a heterogeneous disease requiring specialized treatment [13]. Within the ODAP, a group of experts performed case-by-case evaluations and created a personalized treatment plan—a key a strength of this protocol. Only patients with an inadequate response to optimized conventional treatments were eligible for lumasiran. In ODAP, lumasiran was not given in 24% of PH1 patients (4 of 17 patients with definitive decision). All patients were reviewed by multiple experts per protocol, enhancing data reliability and distinguishing this protocol from standard expert centre monitoring. Continued vigilance allowed revisions to therapeutic advice, as seen in one initially refused patient. The ODAP allowed for a personalized approach with close monitoring over time, providing reliable information on the biochemical and clinical response to lumasiran in real-world setting.

### ODAP as model for optimal management of rare kidney diseases

The adverse outcomes of PH1 are partly due to delayed diagnosis and suboptimal management [14, 15]. The main causes are inadequate follow-up of patients and a general lack of specific knowledge about PH1 among nephrologists. In ODAP, patients are closely followed and discussed by a group of experts in the field of PH1, in close collaboration with the local treating nephrologist or paediatrician. Several patients expressed feeling secure with the close monitoring of kidney function and oxalate levels within ODAP. This network structure contributes to high quality PH1-specific care and increases the general knowledge of PH1 among physicians.

### Cost reduction

In addition to patient benefits, this protocol has potential societal benefits. The ODAP ensures that only patients with an unmet need and expected significant clinical benefit are treated with lumasiran. This contrasts with other countries with access to lumasiran, such as the USA where less stringent protocols apply [2]. In these countries, all patients with PH1 are eligible for treatment, regardless of pyridoxine responsiveness. The strict eligibility criteria within the ODAP ensured that this expensive treatment was administered only to patients with a strong indication. As a result, three patients were denied treatment, leading to an estimated cost reduction of over €1.7 million per year [16]. Furthermore, an additional €1.5 million can be saved each year by minimizing, altogether resulting in a significant cost reduction.

### Guaranteed monitoring of effectivity

The ODAP also secured insight and monitoring in the use of this expensive therapy. Without this national protocol, monitoring of lumasiran use would have been limited, as many physicians would have been eligible to prescribe the drug, as seen in many other European countries [2]. As a result, expertise on use and therapeutic effects would be scattered, minimizing would not be possible and patient enrolment in registries might have been hampered. By requiring data monitoring of all patients treated with lumasiran, high quality data could be collected, further enhanced by monitoring all patients within the same medical centre. All assessments were performed in the same central laboratory, which strengthens the prospective data collected within this protocol as measurements may vary per laboratory, providing reliable and comparable data, like a phase 4 trial. Besides, experience with oxalate and glycolate levels is required for interpretation due to analytical challenges [5]. It is therefore important that the use of lumasiran is monitored by experts in the field.

### New knowledge

At the time of the approval for lumasiran for the treatment of PH1 patients by the EMA, data on clinical effectiveness in PH1 were scarce. Also, the impact of the drug in pyridoxine responsive PH1 patients was not fully understood. So far, we learned that several, but not all patients with pyridoxine responsive variants may respond to pyridoxine to an extent where lumasiran does not add a beneficial reduction in oxalate production. Lumasiran showed a biochemical effect in all patients treated, although only three of the nine patients with eGFR >30 mL/min/1.73 m^2^ had a complete normalization of urinary oxalate levels. However, the effects observed in ODAP are consistent with the results of the extended ILLUMINATE-A trial [17] and no differences in follow-up outcomes were noted between the trial or ODAP. We also learned that lumasiran can be used as bridge therapy to liver transplantation in cases of good but incomplete response to lumasiran. Additional data will be collected and analysed during the remainder of the 5-year ODAP pilot, which will include additional clinical endpoints.

### Limitations

The ODAP for lumasiran showed benefits for patient care, but also limitations. One of the main clinical limitations was that patients had to adhere to the protocol and strict planning of outpatient visits to cluster patients in order to avoid wastage of lumasiran. In addition, from the physician's perspective, participating in ODAP was time-consuming, but undoubtedly considered important to gain national support for the protocolized use of lumasiran in PH1 patients.

### Prospective and novel therapeutics

Besides lumasiran, other novel therapeutics for PH are about to enter the commercial market. For example, nedosiran (Novo Nordisk), has been approved by the FDA and is commercially available in the USA [18]. It is expected to be available on the European market sometime soon. Other novel therapeutics like CRISPR-Cas gene therapy or selective lactate dehydrogenase and glycolate oxidase inhibitors may become available in the future [19, 20]. The pilot of the current ODAP trajectory could be used as a case study to learn lessons for these future novel therapeutics and could be used to integrate real-world data collection in early stages. In the future, it would be interesting to gain further insight into cost-effective dosing regimens for lumasiran.

### Conclusions and recommendations

The ODAP lumasiran pilot proved to be a successful format for optimal rare disease care and efficient and personalized use of expensive orphan drugs in patients with PH1. It also provided additional information on the effectiveness of lumasiran in PH1 patients under specific conditions and can serve as an independent post-marketing surveillance that may lead to an adjustment of the indication. Finally, this ODAP has increased the general knowledge of PH1 among participating physicians. We therefore believe that this model may serve as an example for future access routes to other new expensive orphan therapeutics in the field of rare diseases.

## Supplementary Material

gfaf060_Supplemental_File

## Data Availability

The data underlying this article are available in the article and in its online supplementary material.

## References

[1] Garrelfs SF, Frishberg Y, Hulton SA et al. Lumasiran, an RNAi therapeutic for primary hyperoxaluria type 1. N Engl J Med 2021;384:1216–26. 10.1056/NEJMoa2021712 33789010

[2] Deesker LJ, Oubram L, Almardini R et al. Global access to management of primary hyperoxaluria: a survey on behalf of OxalEurope, G&K Working Group of the ERA and ESPN. Nephrol Dial Transplant 2025; gfaf035. 10.1093/ndt/gfaf035 PMC12394131 39984743

[3] Boon W, Martins L, Koopmanschap M. Governance of conditional reimbursement practices in The Netherlands. Health Policy 2015;119:180–5. 10.1016/j.healthpol.2014.10.013 25467790

[4] Pisters-van Roy A, Barjesteh van Waalwijk van Doorn-Khosrovani S, Reparon-Schuijt C et al. Veelbelovende niet-oncologische weesgeneesmiddelen sneller bij patiënt. Pharm Weekbl 27 **en 28; 05-07-2024**.

[5] Groothoff JW, Metry E, Deesker L et al. Clinical practice recommendations for primary hyperoxaluria: an expert consensus statement from ERKNet and OxalEurope. Nat Rev Nephrol 2023;19:194–211. 10.1038/s41581-022-00661-1 36604599

[6] Milliner DS, Mcgregor TL, Thompson A et al. End points for clinical trials in primary hyperoxaluria. Clin J Am Soc Nephrol 2020;15:1056–65. 10.2215/CJN.13821119 32165440 PMC7341772

[7] Metry EL, Garrelfs SF, Peters-Sengers H et al. Plasma oxalate and glycolate concentrations in dialysis patients with and without primary hyperoxaluria type 1. Nephrol Dial Transplant 2023;38:1773–5. 10.1093/ndt/gfad049 36898675 PMC10310499

[8] Michael M, Groothoff JW, Shasha-Lavsky H et al. Lumasiran for advanced primary hyperoxaluria type 1: phase 3 ILLUMINATE-C trial. Am J Kidney Dis 2023;81:145–55.e1. 10.1053/j.ajkd.2022.05.012 35843439

[9] Metry EL, Deesker LJ, Garrelfs SF et al. Successful kidney-alone transplantation in a patient with PH1 on combination RNA-interference therapy. Kidney Int 2023;104:203–4. 10.1016/j.kint.2023.04.009 37349052

[10] Zamora B, Maignen F, O'Neill P et al. Comparing access to orphan medicinal products in Europe. Orphanet J Rare Dis 2019;14:95. 10.1186/s13023-019-1078-5 31053154 PMC6499954

[11] NICE . Overview | Lumasiran for Treating Primary Hyperoxaluria Type 1 | Guidance | NICE. Available at: https://www.nice.org.uk/guidance/hst25 ( 13 April 2025, date last accessed). 40258103

[12] FAGG . Lumasiran | FAGG. Available at: https://www.fagg.be/nl/MENSELIJK_gebruik/geneesmiddelen/geneesmiddelen/onderzoek_ontwikkeling/gebruik_in_schrijnende_127 ( 13 April 2025, date last accessed).

[13] Deesker LJ, Karacoban HA, Metry EL et al. Intrafamilial disease heterogeneity in primary hyperoxaluria type 1. Kidney Int Rep 2024;9:3006–15. 10.1016/j.ekir.2024.07.026 39430166 PMC11489452

[14] Metry EL, Garrelfs SF, Deesker LJ et al. Determinants of kidney failure in primary Hyperoxaluria type 1: findings of the European Hyperoxaluria Consortium. Kidney Int Rep 2023;8:2029–42. 10.1016/j.ekir.2023.07.025 37849991 PMC10577369

[15] Pszczolinski R, Acquaviva C, Berrahal I et al. Primary hyperoxaluria in adults and children: a nationwide cohort highlights a persistent diagnostic delay. Clin Kidney J 2024;17:sfae099. 10.1093/ckj/sfae099 38737343 PMC11087826

[16] Gang X, Liu F, Mao J. Lumasiran for primary hyperoxaluria type 1: what we have learned? Front Pediatr 2022;10:1052625. 10.3389/fped.2022.1052625 36704142 PMC9871624

[17] Saland JM, Lieske JC, Groothoff JW et al. Efficacy and safety of lumasiran in patients with primary hyperoxaluria type 1: results from a phase III clinical trial. Kidney Int Rep 2024;9:2037–46. 10.1016/j.ekir.2024.04.048 39081738 PMC11284403

[18] Syed YY . Nedosiran: first approval. Drugs 2023;83:1729–33. 10.1007/s40265-023-01976-4 38060091 PMC10803381

[19] Bacchetta J, Lieske JC. Primary hyperoxaluria type 1: novel therapies at a glance. Clin Kidney J 2022;15:i17–22. 10.1093/ckj/sfab245 35592618 PMC9113449

[20] Jiang Y, Chen S, Hsiao S et al. Efficient and safe in vivo treatment of primary hyperoxaluria type 1 via LNP-CRISPR-Cas9-mediated glycolate oxidase disruption. Mol Ther 2025;33:104–18. 10.1016/j.ymthe.2024.10.003 39385468 PMC11764414

[21] Ministerie van Volksgezondheid, Welzijn en Sport. (2025) SAES, Investigators | The Central Committee on Research Involving Human Subjects. Available at: https://english.ccmo.nl/investigators/during-and-after-the-research/saes-and-susars (16 February 2025, date last accessed).

