# Supplementary material for: Controlled access to lumasiran in primary hyperoxaluria type 1: evaluation of a new access route for orphan drugs in the Netherlands
Source: Nephrol Dial Transplant. 2025 Mar 22;40(10):1887–96. doi: 10.1093/ndt/gfaf060 (PMC12477465; doi:10.1093/ndt/gfaf060)
Supplement: gfaf060_Supplemental_File [file gfaf060_supplemental_file.docx]

**Supplementary materials**

**Supplementary table S1. General information on drug, dosing and pricing***

| **Treatment** | **Form and concentration** | **List price** | **Recommended dosage** |
| --- | --- | --- | --- |
| Lumasiran (Oxlumo) | Vials of 0.5 ml containing 94.5 mg | 74 015 euro | ≤ 10 kg: loading dose of 6 mg/kg monthly for 3 doses and a maintenance dose of 3 mg/kg monthly  10 kg to 20 kg: loading dose of 6 mg/kg monthly for 3 doses and a maintenance dose of 6 mg/kg once every 3 months  ≥ 20 kg: loading dose of 3 mg/kg monthly for 3 doses and a maintenance dose of 3 mg/kg once every 3 months |

*Information (in Dutch) may be accessed via: [lumasiran (farmacotherapeutischkompas.nl)](https://www.farmacotherapeutischkompas.nl/bladeren/preparaatteksten/l/lumasiran)

**Supplementary table S2.**

|  | **Pediatric** | **Adult** | **Total** |
| --- | --- | --- | --- |
| **List price** | 74 015euro / 0.5 ml | 74 015 euro / 0.5 ml | 74 015 euro / 0.5 ml |
| **Estimated dosing weight** | 22kg | 89kg | n.a. |
| **Dosing** | 3 mg/kg/quarterly, first 3 months monthly, 0.35 ml per dose | 3 mg/kg/quarterly, first 3 months monthly, 1.3 ml per dose | n.a. |
| **Price per dose** | 51 811 euro | 192 439 euro |  |
| **Price per year (first year, 6 dosages)** | 310 866 euro | 1 154 634 euro | 2 620 134 euro |
| **Price per year (subsequent years, 4 dosages)** | 207 244 euro | 769 756 euro | 1 746 756 euro |
| **Rejection after how many months start ODAP** | 16.3 months | 12.7 and 6.0 months | n.a. |
| **Costs saved during 2 year ODAP** | 259 055* euro | 1 154 634 and 1 539 512 euro | 2 953 201 euro |

*Total reduced price calculated based upon one child of 22 kg and 2 adults with an estimated weight of 82 kg that were not started on lumasiran within the ODAP. Costs are based upon 0% spillage and numbers are rounded. *Based on 5 doses in this period for the pediatric patient and 6 and 8 for the adult patients. Calculated prices are verified with data from the Canadian Agency for Drugs and Technologies in Health (CADTH), which describe costs of 581 132 and 387 421 Canadian dollars for the first respectively subsequent years for a child and 1 743 395 and 1 162 263 Canadian dollars for the first respectively subsequent years for an adult PH1 patient (including spillage). CADTH document may be accessed via:* [Lumasiran (Oxlumo) (cda-amc.ca)](https://www.cda-amc.ca/sites/default/files/DRR/2023/SR0734-Oxlumo.pdf).

**Supplementary table S3**

| **Total vials needed without combining per 3 months** | **Total ml / vials lumasiran of all patients combined per 3 months** | **Adjusted vials needed per 3 months assuming incomplete combating of spillage** | **List price** | **Estimated reduction of costs** |
| --- | --- | --- | --- | --- |
| 33 vials | 14.65 ml / 26.6 vials* | 28 vials | 74 015 euro | Per 3 months: 370 077 euro  Per year: 1 480 310 euro |

**By combining vials, more than the 0.5 ml can be retrieved from one vial leading to a reduced number of needed vials. All numbers are rounded.*

**Supplementary methods S3**

| *Rationale multiple dose vial sharing protocol*  The package leaflet states that the lumasiran vial, once opened should be used immediately and therefore is for single use. A pharmacist at Amsterdam UMC with expertise in minimizing medication wastage, performed a review of lumasiran. It was advised that vial sharing was safe under the conditions that aseptic procedures were used and that vials were not opened for more than 24 hours.  *Medication preparation procedures*  The medication preparation process commenced with the performance of proper hand hygiene, followed by the careful arrangement of necessary supplies, including sterile syringes, withdrawal needles, and injection needles. The medication and patient details were thoroughly verified, ensuring the expiration date, color, and consistency of the medication were appropriate.  Sterile syringes and single-use sterile needles were employed for drawing the medication. Each vial was disinfected with an medical grade 70% alcohol-soaked gauze pad before each needle puncture to maintain sterility and minimize contamination risk. A corresponding amount of air was drawn into the syringe, based on the prescribed dose. The withdrawal needle was then inserted through the vial's rubber stopper, and air was expelled into the vial. The vial was inverted, and the prescribed medication dose was drawn into the syringe.  After the prescribed dose was withdrawn, the syringe was carefully removed from the vial. Air bubbles in the syringe were eliminated by holding the syringe vertically, tapping it gently to move the bubbles upward, and slowly expelling the air. The withdrawal needle was then discarded in a designated sharps container, and the injection needle was attached to the syringe.  Following the preparation, all materials were disposed of appropriately, and hand hygiene was performed once again to ensure the sterility of the preparation process.  For each needle puncture, the above procedures are repeated using clean, sterile materials. Lumasiran vials are not used more than 24 hours after opening (i.e. in practice vials are not used after more than 6 hours). |
| --- |
